# Capturing Human Interaction in the Virtual Age: A Perspective on the Future of fNIRS Hyperscanning

**DOI:** 10.3389/fnhum.2020.588494

**Published:** 2020-11-03

**Authors:** Stephanie Balters, Joseph M. Baker, Grace Hawthorne, Allan L. Reiss

**Affiliations:** ^1^Department of Psychiatry and Behavioral Sciences, School of Medicine, Stanford University, Stanford, CA, United States; ^2^Hasso Plattner Institute of Design, Stanford University, Stanford, CA, United States; ^3^Department of Radiology, School of Medicine, Stanford University, Stanford, CA, United States; ^4^Department of Pediatrics, School of Medicine, Stanford University, Stanford, CA, United States

**Keywords:** hyperscanning, functional near-infrared spectroscopy, fNIRS, social interaction, virtual interaction

## Abstract

Advances in video conferencing capabilities combined with dramatic socio-dynamic shifts brought about by COVID-19, have redefined the ways in which humans interact in modern society. From business meetings to medical exams, or from classroom instruction to yoga class, virtual interfacing has permeated nearly every aspect of our daily lives. A seemingly endless stream of technological advances combined with our newfound reliance on virtual interfacing makes it likely that humans will continue to use this modern form of social interaction into the future. However, emergent evidence suggests that virtual interfacing may not be equivalent to face-to-face interactions. Ultimately, too little is currently understood about the mechanisms that underlie human interactions over the virtual divide, including how these mechanisms differ from traditional face-to-face interaction. Here, we propose functional near-infrared spectroscopy (fNIRS) hyperscanning—simultaneous measurement of two or more brains—as an optimal approach to quantify potential neurocognitive differences between virtual and in-person interactions. We argue that increased focus on this understudied domain will help elucidate the reasons why virtual conferencing doesn't always stack up to in-person meetings and will also serve to spur new technologies designed to improve the virtual interaction experience. On the basis of existing fNIRS hyperscanning literature, we highlight the current gaps in research regarding virtual interactions. Furthermore, we provide insight into current hurdles regarding fNIRS hyperscanning hardware and methodology that should be addressed in order to shed light on this newly critical element of everyday life.

## 1. Introduction

The COVID-19 pandemic has dramatically disrupted the daily lives of much, if not all, of the world's population. Overnight, in-person social interactions have been replaced by video conferencing. Today “Zoom meetings” are commonplace and have largely allowed us to continue engaging in our daily routines. Indeed, in the weeks after COVID-19 emerged across the globe downloads of videoconferencing apps increased by >90% of the 2019 average (AppAnnie.com 2020). Since then, videoconferencing has been a vital tool for business, medicine, education, and social interactions alike. Despite our ability to stay “connected,” there is both empirical and anecdotal evidence to suggest that these mediums are inadequate substitutes for traditional in-person social interactions. For example, virtual interactions have been shown to have adverse effects on emotional and mental health (Holmes et al., 2020; Pfefferbaum and North, 2020), education outcome (Ahmed et al., 2020; Schwartz et al., 2020), and medical care service (Hollander and Carr, 2020; Pappot et al., 2020). Moreover, a glut of popular press articles lamenting the negative effects of “Zoom fatigue” in its many forms (BBC April 22, 2020; National Geographic, April 24, 2020; New York Times, May 4, 2020; the Wall Street Journal, June 5, 2020) are testimony to the negative impact that this new form of communication may have on human-to-human interaction.

These reports are concerning given that video conferencing is likely to play a significant role in human's lives for the foreseeable future (Van Bavel et al., 2020). Critically, too little is currently understood about the underlying neurocognitive mechanisms that result in the adverse effects reported above (e.g., increase in social isolation, decrease in learning outcome, increase in fatigue, etc.). In fact, to our knowledge, there is currently no study that directly compares the differences in neural signatures of social interactions between virtual and in-person interactions. We argue that it is critically important to understand these differences in neural mechanisms that underlie digital human-to-human interaction, and specifically how these neural mechanisms may differ from traditional in-person interactions. We propose functional near-infrared spectroscopy (fNIRS) hyperscanning (i.e., measuring two or more brains simultaneously as they interact socially) as a tool to quantify and understand the potential differences between virtual and in-person interactions. As we argue below, fNIRS hyperscanning may provide an ideal approach to elucidate the neurocognitive differences between virtual and in-person interactions that may result from changes in social behavior (e.g., eye-to-eye contact), from differences in environmental information (e.g., disparate background/foreground lighting), and/or from technological parameters (e.g., unequal frame rates). A clear understanding of the underlying neural mechanisms could inform the development of behavioral interventions and/or the design and engineering of technology that help to mitigate adverse effects. For example, imagine brief yet highly-effective pro-social behavioral exercises that combat social isolation or software that simply synchronizes frame rates to decrease fatigue during virtual teaching/learning activities.

There is conceptual and empirical evidence that social cognition is fundamentally different when we are in interaction with others rather than merely observing them (Schilbach et al., 2013). Hyperscanning technology has allowed us to shed light onto the neural processes underpinning social cognition (Babiloni and Astolfi, 2014; Wang et al., 2018). Over the past decade the field of hyperscanning with functional near-infrared spectroscopy has increased dramatically and has provided unique insight into signatures of brain-to-brain connectivity that are invisible to the naked eye (Dumas et al., 2011; Babiloni and Astolfi, 2014; Redcay and Schilbach, 2019). Specifically, fNIRS hyperscanning has highlighted inter-brain coherence (i.e., correlation of cortical activity between brains) that occurs during social interactions, such as cooperation (Cui et al., 2012; Yang et al., 2020), and is often associated with enhanced behavioral metrics of interaction (Baker et al., 2016). Importantly, given fNIRS' relatively robust tolerance to movement and methodological flexibility, hyperscanning in this modality allows researchers to observe the neural correlates of shared human neural activity in naturalistic environments that are often not feasible in other modalities, such as fMRI or EEG (Scholkmann et al., 2013; Baker et al., 2017; Quaresima and Ferrari, 2019; Gvirts and Perlmutter, 2020). The dramatic increase in fNIRS hyperscanning research has spurred the publication of several systematic reviews, to which we refer the interested reader (Babiloni and Astolfi, 2014; Wang et al., 2018; Czeszumski et al., 2020). In this paper, we focus on providing a review of methodology used in fNIRS hyperscanning research and provide a novel framework to help guide the development of future studies for advancing the field toward capturing human interaction in the virtual age.

## 2. Deriving an fNIRS Hyperscanning Framework

We executed a keyword search via Google Scholar and PubMed up to May 15, 2020 that included the following keywords: “fNIRS hyperscanning” and “NIRS hyperscanning.” For each search engine, we inspected the first 250 entries for each keyword category and checked the reference lists of the included articles for any additional relevant articles. We included journal and conference articles in the English language only, resulting in a total of 69 fNIRS hyperscanning studies. For the scope of this paper, we focused only on those studies that investigated interaction between adults. As such, we excluded nine infant-parent fNIRS hyperscanning studies (Leong et al., 2017; Reindl et al., 2018; Azhari et al., 2019, 2020; Miller et al., 2019; Quiñones-Camacho et al., 2019; Behrendt et al., 2020; Nguyen et al., 2020; Piazza et al., 2020). Furthermore, we excluded two papers that included comparisons of temporally non-congruent fNIRS scans (Liu Y et al., 2017; Hou et al., 2020), resulting in a total of 58 fNIRS hyperscanning papers (see Table 1 for an overview). From each of the resulting 58 fNIRS hyperscanning papers, we extracted all experimental conditions (i.e., “hyperscan” conditions) that were utilized and from which data were analyzed.

**Table 1:** List of 55 fNIRS hyperscanning studies—updated APRIL 15 2020.

**References**	**Setup**	**Transfer of information**	**Transfer of communication**	**Cognitive function**	**Region**
Funane et al. (2011)	6 dyads, 1 scan cond.	Analog (FtF button press)	Joint goal-driven (cooperation)	Attention* Executive function* Motor	PFC
Cui et al. (2012)^WTC^	11 dyads (2ff,8fm,1mm), 4 scan cond.	Mixed (SbS computer task)	Joint goal-driven (cooperation, competition) and mixed (congruent action-observe)	Attention* Executive function* Motor	PFC
Dommer et al. (2012)^WTC^	4 dyads, 1 scan cond.	Mixed (SbS computer task)	Joint goal-driven (cooperation)	Attention* Executive function* Motor	lPFC
Holper et al. (2012)^WTC^	8 dyads, 2 scan cond.	Analog (FtF synchronization task)	Joint goal-driven (cooperation)	Attention Executive function Motor*	pMC
Jiang et al. (2012)^WTC^	10 dyads (6ff,4mm), 6 scan cond.	Analog (FtF vs. BtB verbal task)	Joint goal-driven (cooperation, competition) and mixed (congruent action-observe)	Attention* Executive function* Language*	lPFC lTC lPC
Duan et al. (2013)	1 dyad (mm), 1 scan cond.	Mixed (SbS computer task)	Joint goal-driven	Attention Executive function Motor* Visuospatial function	lMC
Holper et al. (2013)	17 dyads, 4 scan cond.	Analog (FtF verbal task)	Joint goal-driven (cooperation, competition) and mixed (congruent action-observe)	Attention* Executive function* Memory Language	lPFC
Osaka et al. (2014)^WTC^	14 dyads (5ff,9mm), 4 scan cond.	Analog (FtF vs. SbS singing task)	Joint goal-driven (cooperation, competition) and mixed (congruent action-observe)	Attention* Executive function* Language*	Whole head
Cheng et al. (2015)^WTC^	45 dyads (15ff,16fm,14mm), 4 scan cond.	Mixed (SbS computer task)	Joint goal-driven (cooperation, competition) and mixed (congruent action-observe)	Attention* Executive function* Motor	PFC
Duan et al. (2015)	1 nonad, 1 scan cond.	Analog (FtF music instrument task)	Joint goal-driven (cooperation)	Attention* Executive function* Motor	mPFC lPC(TPJ)
Jiang et al. (2015)^WTC^	12 triads (6fff, 6mmm), 1 scan cond.	Analog (FtF verbal task)	Joint goal-driven	Attention* Executive function* Language* Social cognition*	lPFC lPC(TPJ)
Liu T et al. (2015)	10 dyads, 4 scan cond.	Mixed (SbS computer task)	Joint goal-driven (cooperation, competition) and mixed (congruent action-observe)	Attention* Executive function* Motor Visuospatial function	lPFC lPC(TPJ)
Osaka et al. (2015)^WTC^	15 dyads (7ff, 8mm), 4 scan cond.	Analog (FtF vs. FtF-shielded singing task)	Joint goal-driven (cooperation, competition) and mixed (congruent action-observe)	Attention* Executive function* Language*	Whole head
Baker et al. (2016)^WTC^	111 dyads (38ff,34fm,39mmf), 1 scan cond.	Mixed (FtF computer task)	Joint goal-driven (cooperation)	Attention* Executive function* Motor	lPFC rPC(TPJ)
Liu N et al. (2016)^WTC^	9 dyads (2ff, 5fm, 2mm), 4 scan cond.	Analog (FtF Jenga game)	Joint goal-driven (cooperation, congruent action-action) and joint open-ended	Attention* Executive function* Motor Language Visuospatial function	rPFC rTC
Liu T et al. (2016)	10 dyads (2ff, 8 mm), 4 scan cond.	Mixed (SbS computer task)	Joint goal-driven (cooperation, competition) and mixed (congruent action-observe)	Attention* Executive function* Motor Visuospatial function	PFC
Nozawa et al. (2016)^WTC^	12 quartets, 2 scan cond.	Analog (FtF/SbS verbal task)	Joint goal-driven (cooperation)	Attention* Executive function* Language	mPFC
Tang et al. (2016)^WTC^	101 dyads, 2 scan cond.	FtF computer task	Joint goal-driven	Attention* Executive function* Social cognition*	mPFC rPC(TPJ)
Balconi and Vanutelli (2017a)	16 dyads, 2 scan cond.	Mixed (SbS-shielded computer task)	Joint goal-driven (cooperation)	Attention* Executive function* Social cognition	PFC
Balconi and Vanutelli (2017b)	14 dyads, 2 scan cond.	Mixed (SbS-shielded computer task)	Joint goal-driven (cooperation)	Attention* Executive function* Motor Social congition	PFC
Hirsch et al. (2017)^WTC^	19 dyads (6ff, 10fm, 3mm), 2 scan cond.	Analog (FtF, visual/ non-verbal task)	Joint goal-driven (cooperation)	Attention* Executive function*	PFC PC TC
Hu et al. (2017)^WTC^	35 dyads (all ff) 2 scan cond.	Mixed (FtF-shielded computer task)	Joint goal-driven (cooperation)	Attention* Executive function* Motor	PFC
Ikeda et al. (2017)^WTC^	4 groups of 24 or 25, 4 scan cond.	Analog (FtB and BtB synchronization task)	Joint goal-driven (cooperation) and joint open-ended	Attention* Executive function* Motor	mPFC
Liu T et al. (2017)	22 dyads (all mm) 4 scan cond.	Mixed (SbS computer task)	Joint goal-driven (cooperation, competition) and mixed (congruent action-observe)	Attention* Executive function* Motor Visuospatial function	PC
Pan et al. (2017)^WTC^	49 dyads (all fm) 1 scan cond.	Mixed (FtF-shielded computer task)	Joint goal-driven (cooperation)	Attention* Executive function* Motor	rPFC rPC
Piva et al. (2017)^WTC^	20 dyads (4ff, 16fm, 2mm), 2 scan cond.	Mixed (FtF computer task)	Joint goal-driven (competition)	Attention* Executive function* Motor Language* Social cognition*	PFC PC
Takeuchi et al. (2017)	15 dyads (4ff, 3fm,8mm), 1 scan cond.	Mixed (SbS computer task)	Joint goal-driven (cooperation)	Attention* Executive function* Motor Visuospatial cognition Social cognition	PFC
Zhang et al. (2017a)^WTC^	30 dyads, 2 scan cond.	Analog (FtF card game)	Joint goal-driven (cooperation, competition)	Attention* Executive function* Motor Language* Visuaspatial cognition Social cognition*	mPFC lPFC lPC(TPJ)
Zhang et al. (2017b)^WTC^	33 dyads, 2 scan cond.	Analog (FtF card game)	Joint goal-driven (cooperation, competition)	Attention* Executive function* Motor Language* Visuaspatial cognition Social cognition*	mPFC lPFC lPC(TPJ)
Zhao et al. (2017)	48 dyads (24ff, 24mm), 3 scan cond.	Analog (BtB music instrument task)	Joint goal-driven (cooperation)	Attention* Executive function* Motor	mPFC rPFC
Dai et al. (2018a)^WTC^	48 dyads (24ff, 24mm), 3 scan cond.	Analog (BtB music instrument task)	Joint goal-driven (cooperation)	Attention* Executive function* Motor	lPFC lPC lTC
Dai et al. (2018b)	22 triads (all same sex), 4 scan cond.	Analog (FtF and BtB verbal task)	Joint goal-driven (cooperation)	Attention* Executive function* Language*	lPFC lPC lTC
Fishburn et al. (2018)	20 triads, 5 scan cond.	Analog (FtF Tangram puzzle) and mixed (watching movie)	Joint goal-driven (cooperation, competition) and mixed (congruent action-observe) and joint-open ended (watch movie)	Attention* Executive function* Motor Language Visuospatial function	rPFC lPFC
Hirsch et al. (2018)^WTC^	27 dyads (10ff,12fm,5mm), 4 scan cond.	Analog (FtF-shielded verbal task)	Joint goal-driven (cooperation, competition) and mixed (congruent action-observe)	Attention* Executive function* Language*	PFC PC
Pan et al. (2018)^WTC^	12 dyads, 2 scan cond.	Analog (FtF singing task)	Joint goal-driven (cooperation)	Attention* Executive function* Memory Language*	lPFC lPC lTC
Xue et al. (2018)^WTC^	45 dyads, 1 scan cond.	Analog (FtF verbal task)	Joint goal-driven (cooperation)	Attention* Executive function* Social cognition*	PFC rPC(TPJ)
Zhang Y et al. (2018)^WTC^	17 dyads, 2 scan cond.	Analog (FtF verbal task)	Joint goal-driven (cooperation) and joint open-ended	Attention* Executive function* Language Memory Social cognition*	PFC rPC(TPJ)
Zhang M et al. (2018)^WTC^	31 dyads, 1 scan cond.	Mixed (SbS computer task)	Joint goal-driven (cooperation)	Attention* Executive function* Social cognition	PFC
Zheng et al. (2018)^WTC^	32 dyads, 2 scan cond.	Mixed (SbS computer task)	Joint goal-driven (cooperation)	Attention* Executive function* Language* Memory Social cognition*	PFC PC TC
Balconi et al. (2019)	31 dyads 16 dyads (all ff), 2 scan cond.	Mixed (SbS-shielded computer task)	Joint goal-driven (cooperation)	Attention* Executive function* Memory Social cognition	PFC pMC
Cheng et al. (2019)^WTC^	31 dyads (16ff, 15fm), 2 scan cond.	Mixed (FtF-shielded computer task)	Joint goal-driven (cooperation)	Attention* Executive function* Motor	PFC
Liu et al. (2019)^WTC^	21 dyads, 4 scan cond.	Mixed (FtF vs. BtB computer and verbal task)	Joint goal-driven (cooperation)	Attention* Executive function* Language Memory Social cognition*	PFC rPC(TPJ)
Lu et al. (2019)^WTC^	52 dyads, 4 scan cond.	Analog (FtF verbal task)	Joint goal-driven (cooperation)	Attention* Executive function* Language	PFC rPC(TPJ)
Mayseless et al. (2019)^WTC^	25 dyads (8ff, 8fm,9mm), 2 scan cond.	Analog (FtF verbal and puzzle task)	Joint goal-driven (cooperation)	Attention* Executive function* Language* Memory Motor Social cognition*	lPFC lPC(TPJ) lTC
Niu et al. (2019)	20 dyads (1ff, 9mm), 4 scan cond.	Analog (SbS synchronization task)	Joint goal-driven (cooperation, competition) and mixed (congruent action-observe)	Attention* Executive function* Motor	rPFC rPC
Nozawa et al. (2019)^WTC^	32 dyads (9ff, 23mm), 4 scan cond.	Analog (FtF verbal task) and mixed (FtF synchronization task)	Joint goal-driven (cooperation)	Attention* Executive function* Language Memory Social cognition	PFC
Sarinasadat et al. (2019a)^WTC^	15 dyads, 2 scan cond.	Mixed (FtF computer task)	Joint goal-driven (cooperation)	Attention* Executive function* Language Memory Social cognition	PFC
Sarinasadat et al. (2019b)^WTC^	15 dyads, 2 scan cond.	Mixed (FtF computer task)	Joint goal-driven (cooperation)	Attention* Executive function* Language Memory Social cognition	PFC
Vanzella et al. (2019)	5 dyads, 4 scan cond.	Analog (SbS music instrument task)	Joint goal-driven (cooperation, competition) and mixed (congruent action-observe)	Attention* Executive function* Memory Motor*	dPFC MC TC
Balconi and Fronda (2020)	15 dyads (all ff), 2 scan cond.)	Mixed (SbS-shielded computer task)	Joint goal-driven (cooperation)	Attention* Executive function* Memory Social cognition	PFC pMC
Feng et al. (2020)^WTC^	120 dyads (60ff, 60mm), 2 scan cond.	Mixed (FtF-shielded computer task)	Joint goal-driven (cooperation, and congruent action-action)	Attention* Executive function* Memory Language Social cognition	PFC
Lu et al. (2020)	66 dyads (26ff,22fm,18mm), 2 scan cond.	Analog (FtF verbal task)	Joint goal-driven (cooperation)	Attention* Executive function* Memory Language	PFC rPC(TPJ)
Noah et al. (2020)^WTC^	15 dyads, 2 scan cond.	Analog (FtF non-verbal task) and mixed video watching	Joint goal-driven (cooperation)	Attention* Executive function* Social cognition*	PFC PC
Pan et al. (2020a)^WTC^	24 dyads (all ff), 4 scan cond.)	Analog (FtF verbal task)	Joint goal-driven (cooperation)	Attention* Executive function* Memory Language* Social cognition*	PFC lPC lTC
Pan et al. (2020b)^WTC^	16 dyads (all ff), 1 scan cond.)	Mixed (SbS computer task)	Joint goal-driven (cooperation)	Attention* Executive function* Memory Language* Social cognition*	PFC PC TC
Sun et al. (2020)^WTC^	34 dyads (27ff,7mm), 2 scan cond.	Mixed (FtF-shielded computer task)	Joint goal-driven (cooperation, congruent action-action)	Attention* Executive function* Motor	PFC
Yang et al. (2020)^WTC^	93 sextets (51ffffff,42mmmmmm), 3 scan cond.	Mixed (FtF verbal and computer task)	Joint goal-driven	Attention* Executive function* Memory Motor Language Social cognition*	PFC rPC(TPJ)
Zheng et al. (2020)^WTC^	32 dyads, 2 scan cond.	Mixed (SbS computer task)	Joint goal-driven (cooperation)	Attention* Executive function* Memory Language* Social cognition*	PFC PC TC

ff, female-female; fm, female-male; mm, male-male; FtF, face-to-face; SbS, side-by-side; BtB, back-to-back; PFC, prefrontal cortex; PC, parietal cortex; TC, temporal cortex; l, left; r, right; m, medial; TPJ, temporoparietal junction. Shielded refers to a setup in which participants interaction is shielded by a physical divider, and cond. is the abbreviation for condition(s). We marked those studies that included wavelet coherence analysis “WTC.” We further included cognitive functions that were required to execute the experimental task and highlighted those cognitive functions that were investigated with an “*”.

In order to find a consistent methodological structure across the resulting 151 hyperscans, two researchers (SB and JMB) executed a thematic analysis. Two naturally occurring dimensions (i.e., Transfer of Information and Type of Communication) emerged from each scan. First, Transfer of Information (ToI) refers to the interface through which human-to-human interaction was conveyed. We clustered TOI into three levels: (1) hyperscans that comprised human-to-human interaction in a face-to-face setting (i.e., *Analog*), where no digital medium was present; (2) hyperscans that comprised a combination of analog and digital transfer methods (i.e., *Mixed ToI*), such as sitting side-by-side while problem solving on a computer screen; and (3) hyperscans in which all interactions were made via technology (i.e., *Digital*). Next, *Type of Communication* (ToC) refers to the objective of the interaction and varied between *Joint goal-driven, Joint open-ended*, and *Mixed ToC* interactions. For this classification, any hyperscanning task that had an explicit and clearly defined objective, goal, or competitive outcome (e.g., zero-sum game), or one that compared task accuracy or response time was classified as goal-driven. Conversely, any task that required no explicit objective (e.g., chitchat between therapist and client before the therapy session) was classified as open-ended. Scans that contained elements of both (e.g., two participants cooperate while a 3rd watches) was defined as mixed ToC.

**Figure 1:**
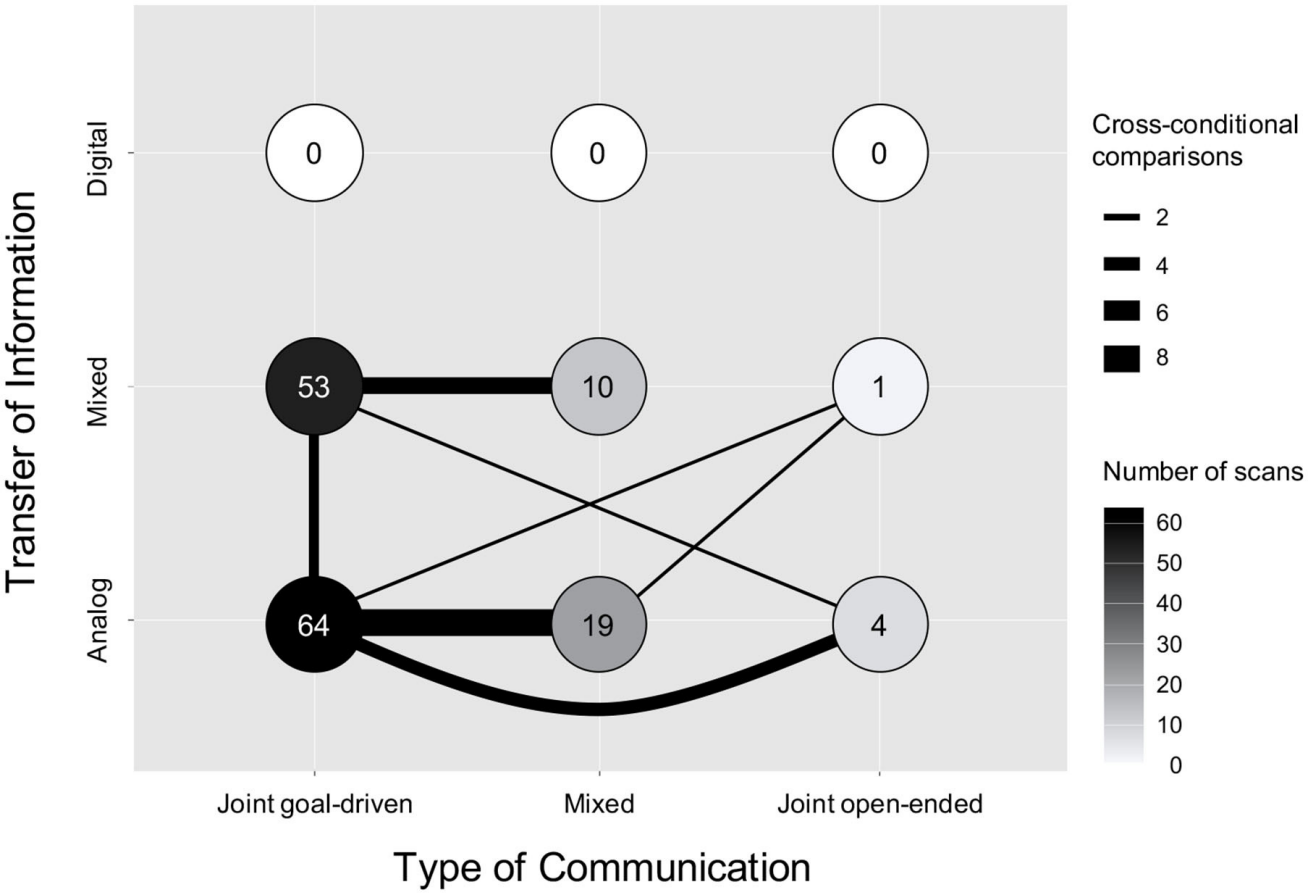
The figure above visualizes the cross-sectional distribution of all 151 conducted hyperscan conditions across the Transfer of Information (ToI) and Type of Communication (ToC) axes. The color of each circle provides the number of scans that belong to each cross-sectional condition. Light colors indicate fewer scans and darker colors indicate more scans. The lines indicate the cross-condition comparisons that were reported. The width of each line provides an illustration of the number of scans conducted within each cross-condition comparison.

As shown in Figure 1, the distribution of hyperscan conditions dedicated to each of the nine categories defined by our framework is highly unequal. Over half of all reported hyperscan conditions (57.8%, *N* = 87) were conducted when the interacting dyad were in the same room without any means of digital interaction (i.e., Analog ToI) (Funane et al., 2011; Holper et al., 2012, 2013; Jiang et al., 2012, 2015; Osaka et al., 2014, 2015; Duan et al., 2015; Liu N et al., 2016; Nozawa et al., 2016, 2019; Hirsch et al., 2017, 2018; Ikeda et al., 2017; Zhang et al., 2017a,b; Zhao et al., 2017; Dai et al., 2018a,b; Fishburn et al., 2018; Pan et al., 2018, 2020a; Xue et al., 2018; Zhang Y et al., 2018; Lu et al., 2019, 2020; Mayseless et al., 2019; Niu et al., 2019; Vanzella et al., 2019; Noah et al., 2020), while (42.4%, *N* = 64) included some element of technology (e.g., playing a computer game) while participants were in the same room (Cui et al., 2012; Dommer et al., 2012; Duan et al., 2013; Cheng et al., 2015, 2019; Liu T et al., 2015, 2016, 2017; Baker et al., 2016; Tang et al., 2016; Balconi and Vanutelli, 2017a,b; Hu et al., 2017; Pan et al., 2017, 2020b; Piva et al., 2017; Takeuchi et al., 2017; Fishburn et al., 2018; Zhang M et al., 2018; Zheng et al., 2018, 2020; Balconi et al., 2019; Liu et al., 2019; Nozawa et al., 2019; Sarinasadat et al., 2019a,b; Balconi and Fronda, 2020; Feng et al., 2020; Noah et al., 2020; Sun et al., 2020; Yang et al., 2020). Notably, no study employed a method in which both interacting members of a dyad were entirely physically separated, such that all interactions were conducted via technology (e.g., virtual meeting). A similar lack of empirical focus currently exists for open-ended ToC. Only 3.3% of all scans (*N* = 5) focused on inter-brain synchrony during open-ended communication (Liu N et al., 2016; Ikeda et al., 2017; Fishburn et al., 2018; Zhang Y et al., 2018; Yang et al., 2020), whereas 77.5% (*N* = 117) focused on Joint goal-driven interactions. The remaining 19.2% (*N* = 29) tasks contained elements of both communication types (Cui et al., 2012; Jiang et al., 2012; Holper et al., 2013; Osaka et al., 2014, 2015; Cheng et al., 2015; Liu T et al., 2015, 2016, 2017; Fishburn et al., 2018; Hirsch et al., 2018; Niu et al., 2019; Vanzella et al., 2019).

The thickness of the lines in Figure 1 represent the frequency of cross-condition comparisons reported. A cross-condition comparison occurred when the ToI or ToC during a hyperscan differed between experimental tasks. A total of 19 (33.3%) papers included in our analysis included one or more cross-condition comparisons. However, the classification of the comparisons reported were limited to *Analog vs. Mixed* and *Inter-ToI* comparisons. The studies comprised comparisons between Mixed ToI/Joint goal-driven ToC and Mixed ToI/Mixed ToC (*N* = 5) (Cui et al., 2012; Cheng et al., 2015; Liu T et al., 2015, 2016, 2017), Analog ToI/Mixed ToC and Analog ToI/Joint goal-driven ToC (*N* = 7) (Jiang et al., 2012; Holper et al., 2013; Osaka et al., 2014, 2015; Hirsch et al., 2018; Niu et al., 2019; Vanzella et al., 2019), Analog ToI/Joint open-ended ToC and Analog ToI/Joint goal-driven ToC (*N* = 3) (Liu N et al., 2016; Ikeda et al., 2017; Zhang Y et al., 2018), and Analog ToI/Mixed ToC and Mixed ToI/Joint goal-driven ToC (*N* = 2) (Nozawa et al., 2019; Noah et al., 2020). Two studies included three cross-condition comparisons, including comparisons between Analog ToI/Mixed ToC, Analog ToI/Joint goal-driven ToC, and Mixed ToI/Joint open-ended ToC (*N* = 1) (Fishburn et al., 2018), as well as between Analog ToI/Joint goal-driven ToC, Analog ToI/Joint open-ended ToC, and Mixed ToI/Joint goal-driven ToC (*N* = 1) (Yang et al., 2020).

## 3. Existing fNIRS Hyperscanning Hurdles

Taken together, our analysis highlights the areas of study that have received little to no attention. Specifically, no fNIRS hyperscanning study has, to date, focused on understanding pure Digital ToI (i.e., virtual meeting) nor has any study focused on comparing Digital ToI with Analog ToI (i.e., in-person meeting). Similarly, Joint open-ended ToC (e.g., chit chat with a friend via zoom) has received very little empirical attention.

The lack of focus on Digital ToI has likely been due, in part, to technological or methodological shortcomings that constrain this line of research. For instance, many fNIRS devices do not easily accommodate a digital hyperscanning interface, which would ostensibly take place in separate rooms so that no in-person communication may occur. While it may be feasible, for example, to build a structure that splits optodes of one device allowing to scan two distant participants, this may be unrealistic for researchers in many instances. Thus, when faced with this challenge, even interested researchers may find such methodology prohibitively difficult. One alternative may be the use of two individual fNIRS devices, each positioned in their own room. However, aside from cost-related drawbacks, in this instance researchers must be able to accurately sync the time series' recorded from both devices in order to facilitate downstream processing and analysis of their data. This may require the development of sophisticated software to sync and timestamp event markers wirelessly across both devices. Notably, while promising examples for such analytical tools do exist (e.g., Labstreaminglayer), there is currently no readily available tool designed specifically for fNIRS hyperscanning. We argue that more effort is needed to develop and disseminate such analytical tools via peer-reviewed publication and open-source file sharing. Alternatively, researchers may video record both members of a separated dyad to capture events, then code the event timestamps *post-hoc*. This procedure is useful but requires a considerable amount of time and manual effort. Moreover, such procedures should be performed in tandem, so that inter-rater reliability may be established. It is our hope that advances within the community will help overcome this hardware hurdle in order to facilitate the study of the digital ToI domain.

The lack of data within the open-ended ToC domain may be less due to technological drawbacks, and more due to a lack of established analytical approaches to tasks that are not trial based. To quantify and analyze brain-to-brain coupling, researchers have applied more traditional statistical approaches, such as block-averaging (e.g., Holper et al., 2013); analysis of co-variance (e.g., Funane et al., 2011); and correlation analysis (e.g., Duan et al., 2013). Cui et al. (2012) introduced a novel analytical approach for fNIRS hyperscanning (i.e., Wavelet Transform Analysis or “WTC”), wherein the coherence and phase lag in two time series is assessed across both time and frequency. By contrasting the average task-related coherence during the task (i.e., cooperation paradigm) and rest, the authors demonstrated an increase in coherence during cooperation that dissipated during rest. Wavelet coherence analysis has been widely adopted within the fNIRS hyperscanning research (as shown in Table 1, roughly 70% of all studies included WTC analysis), and there are efforts to further improve WTC's efficacy (Zhang et al., 2020). However, while the method was originally developed for block-design studies in which a task frequency band and condition markers may be identified, it currently lacks the ability to derive instant and fluctuating components of social interactions. Recent approaches (e.g., Mayseless et al., 2019) have therefore attempted to develop novel analytical methods that do not rely on task blocks, and which may be applicable to open-ended task designs. Finally, Granger Causality, a method that allows for the derivation of directionality of synchrony between two time series, has also been shown to be a useful analytical approach to investigate the fluctuations of interactive dynamics between individuals (Holper et al., 2012). Similar to WTC, further advances in Granger Causality analysis might allow for investigations of fluctuating social dynamics during joint open-ended interactions. It will be important for future research to build upon these approaches, and to develop algorithms and techniques to better facilitate analysis of hyperscanning data.

## 4. A Perspective of the Future Potential of fNIRS Hyperscanning

**Figure 2:**
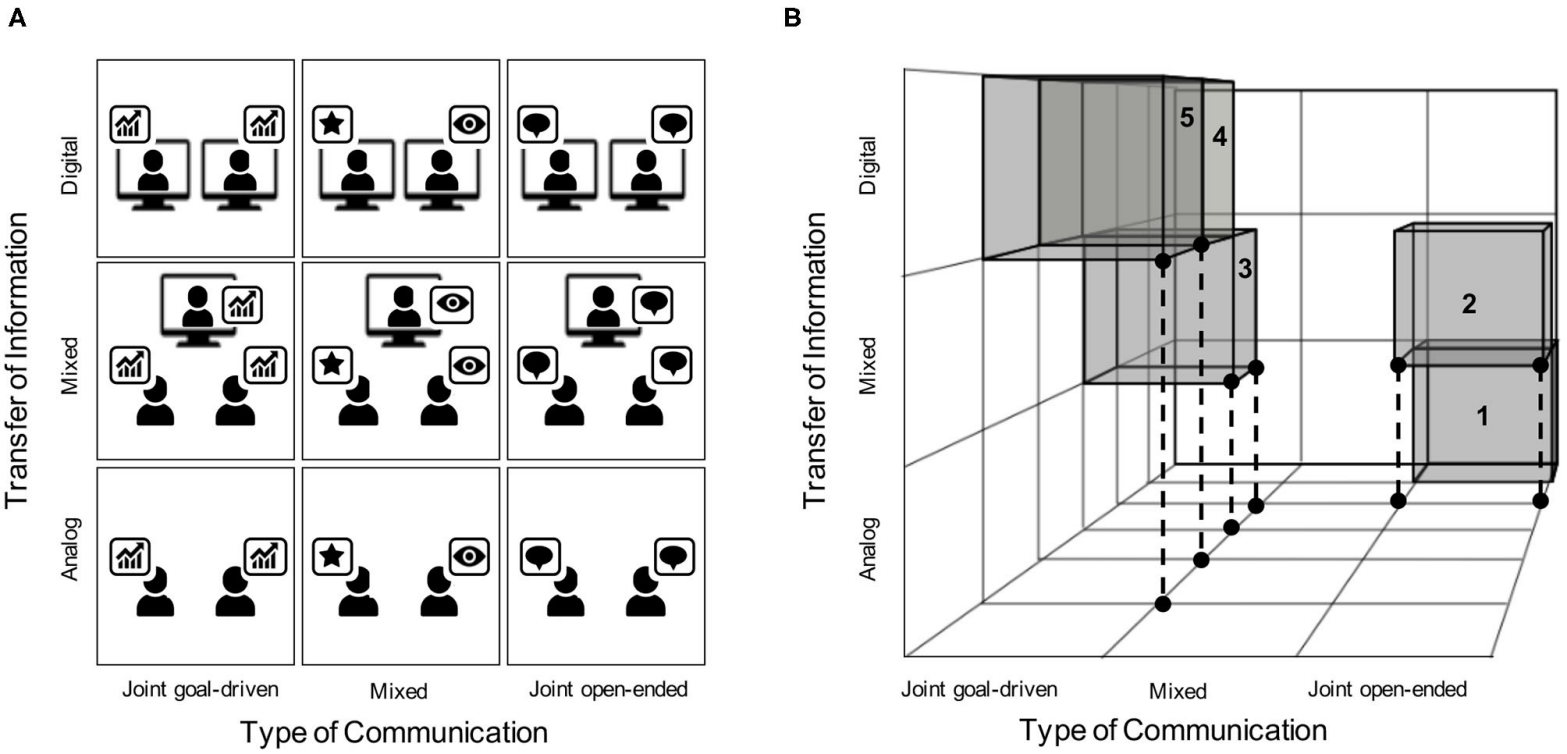
**(A)** This matrix provides a schematic of all nine possible intersections of ToI and ToC within our framework. The schematic shows three hypothetical tasks being conducted across each intersection. First, data analysis (denoted by the bar chart) provides an example of a Joint goal-directed ToC. Next, the instance of one person performing (denoted by the star) while one or more people watch passively (denoted by the eye), provides an example of a Mixed ToC task. Finally, friendly chit-chat (denoted by the chat bubbles) provides an example of a Joint open-ended ToC. Importantly, each of these activities may be conducted under Analog, Mixed, or Digital ToI. **(B)** This schematic demonstrates a hypothetical 3-person hyperscan that fluctuates continuously across time through multiple domains outlined in our framework. First, a pair of participants situated in the same room engage in open-ended conversation for a period of time (1). Next, a third participant joins the pair via a live video feed, which introduces a mixed digital interface between the three participants (2). Following a period of chit-chat, the triad begins work on a goal-driven task together (3). Next, one of the two participants situated together exits, leaving an interacting pair separated by a digital divide that work together on a goal-driven task (4). These participants continue to work on the goal-driven task until completion (5).

The structure presented in Figure 1 is reminiscent of a similar framework that was introduced earlier in this journal (Liu and Pelowski, 2014). Specifically, Liu and Pelowski (2014) proposed a framework that distinguished between task structure (interdependent vs. independent), interaction structure (concurrent vs. turn-based), and goal structure (cooperative vs. competitive) as variables that hyperscanning studies should consider during task design. As the field of fNIRS hyperscanning progresses toward *Real-life Neuroscience* (Shamay-Tsoory and Mendelsohn, 2019; Holleman et al., 2020), the need for an updated framework that includes virtual social interactions (i.e., Digital ToI) as well as open-ended interactions (i.e., Joint open-ended ToC) is warranted. We propose that our updated framework, as depicted in Figure 2A, can help guide hyperscanning researchers toward a future where all forms of human-to-human social interactions are fairly represented. In order to achieve equal distributions, the community has to overcome the current hurdles as described above. These hurdles include, but are not limited to, developing methodological designs that address each condition in Figure 2A, hardware that is amenable to hyperscanning when participants are separated physically, and software that is capable of managing back-end data streams of such tasks. It is our hope that both hardware and software will be flexible enough to approach more and more realistic scenarios in which complex and sudden social interactions can be captured (see Figure 2B).

Efforts from the broader fNIRS community will be required to make fNIRS truly ready for realistic scenarios. With respect to hardware, this includes increased device portability and robustness (e.g., with respect to movement and environmental light), increased optode number to cover more cortical areas, and short-channels to account for extra-cerebellar blood flow that may contaminate fNIRS signals (Brigadoi and Cooper, 2015; Baker et al., 2017; Herold et al., 2017). Furthermore, efforts should be made with respect to standardizing fNIRS procedures, such as optode placement, data processing, choice of activation proxy (i.e., oxy- vs. de-oxygenated hemoglobin) (Brigadoi et al., 2014; Tachtsidis and Scholkmann, 2016; Herold et al., 2017; Di Lorenzo et al., 2019), and adoption of standardized open-source fNIRS-specific data analysis packages (e.g., HOMER2, NIRS SPM, nirsLAB, open-potato, etc.).

While adherence to our framework will help to more completely elucidate the neurobiological signatures of human-to-human interactions across all platforms, future research in this field will not be without limitations. Primarily, this includes the cortical depth at which fNIRS may sample while maintaining acceptable signal quality. While efforts have been made to infer deep-brain activity using fNIRS (Liu N et al., 2015), the relatively low sampling depth of ~3cm (Brigadoi and Cooper, 2015) limits the neurocognitive functions that may be directly measured by fNIRS. As shown in Table 1, the existing fNIRS hyperscanning research has focused on studying cognitive functions within cortical regions underlying attention, executive function, language, social cognition, visuospatial processing, and motor activity. Methodological approaches to the existing fNIRS hyperscanning studies have been diverse and focused on social interactions during simple motor-synching (e.g., Holper et al., 2012), cooperative and competitive gameplay (e.g., Cui et al., 2012), unstructured and structured conversation including singing (e.g., Osaka et al., 2014), teaching activities (e.g., Nozawa et al., 2019), and creative problem solving (e.g., Lu et al., 2019). Studies also tested for effects of moderators, such as sex (Cheng et al., 2015), level of acquaintance (Pan et al., 2017), eye-to-eye contact (e.g., Hirsch et al., 2017), and pro-social priming effects (e.g., Balconi et al., 2019) on inter-brain cognitive functioning and task outcome. In fact, the methodological flexibility afforded by fNIRS is so great that researchers may run the risk of creating methods that are so creative as to be difficult to interpret, replicate or compare. Therefore, we encourage researchers in the immediate future to parsimoniously advance into the understudied areas of our framework (i.e., Digital ToI and JoinT open-ended ToC). For instance, it may be useful to commence the study of differences between virtual and in-person interactions with established hyperscanning tasks, such as simple computer-based cooperation tasks (Cui et al., 2012). In this manner, researchers may directly investigate the effect of ToC on inter-brain coherence and are further able to compare new data with existing outcomes (i.e., confirmatory science). Another interesting inroad could be to extend the study of differences in social cognition between “observing others” and “actually interacting with them” (Schilbach et al., 2013) to video/virtual interactions. In that case, prior fNIRS studies assessing the temporally non-congruent inter-brain coherence of video-recorded individuals and spectators (who watch the videos at a later stage) could serve as entry points (Liu Y et al., 2017; Hou et al., 2020).

Ultimately, multi-dimensional data approaches will allow us to determine which parameters (i.e., behavioral, environmental, and/or technological) are most explanatory with respect to potential differences in neurocognitive signatures between virtual and in-person interactions. For example, using congruent fNIRS-EEG systems will improve temporal resolution. Physiological metrics (e.g., heart rate, heart rate variability, galvanic skin response, pupil dilation, etc.) along with behavioral measures (e.g., eye-gaze-tracking, body-motion tracking, analysis of voice, emotional face tracking, etc.) will provide vital information to better understand the humans' psychophysiological response during social interactions. Lastly, the monitoring of environmental information (e.g., ambient noise, reflecting light on reading glasses, etc.) and technological parameters (e.g., computer frame-rate, computer audio, internet speed, computer screen activity, etc.) will be essential to control and account for potential external biases.

The future of fNIRS hyperscanning is limitless and very well may be a key component of our understanding of the neurobiological underpinnings of social behavior. From tele-health to tele-education, and from internet dating to online gaming, technology driven activities will likely play a ubiquitous role in our social interactions moving forward. The framework presented here is meant to advance discussion among researchers in their study of all aspects of human interaction, including those that technology has yet to make possible.

## Data Availability Statement

All datasets generated for this study are included in the article/supplementary material.

## Author Contributions

SB: conceptualization, literature review, methodology, and writing. JMB: conceptualization, methodology, and writing. GH: conceptualization. ALR: conceptualization, methodology, supervision, and writing. All authors contributed to the article and approved the submitted version.

## Conflict of Interest

The authors declare that the research was conducted in the absence of any commercial or financial relationships that could be construed as a potential conflict of interest.
